# Supplementary material for: Diverse roles of TssA‐like proteins in the assembly of bacterial type VI secretion systems
Source: EMBO J. 2019 Aug 12;38(18):e100825. doi: 10.15252/embj.2018100825 (PMC6745524; doi:10.15252/embj.2018100825)
Supplement: Supplementary file 1 — Appendix [file EMBJ-38-e100825-s001.pdf]

Table of contents

Appendix Figure S1. Photobleaching of cytosolic TssA during sheath polymerization

Appendix Figure S2. Cryo-EM of $TssA_{VC}$

Appendix Figure S3. $TssA_{VC}$ purification

Appendix Figure S4. $TssA_{VC}$ and $TagA_{VC}$ protein interaction assays

Appendix Table S1. TssA proteins pairwise identity (%)

Appendix Table S2. Strains used in this study

Appendix Table S3. Summary of Co-IP and subsequent MS analysis

Appendix Table S4. EM Data collection and processing

Appendix Figure S1

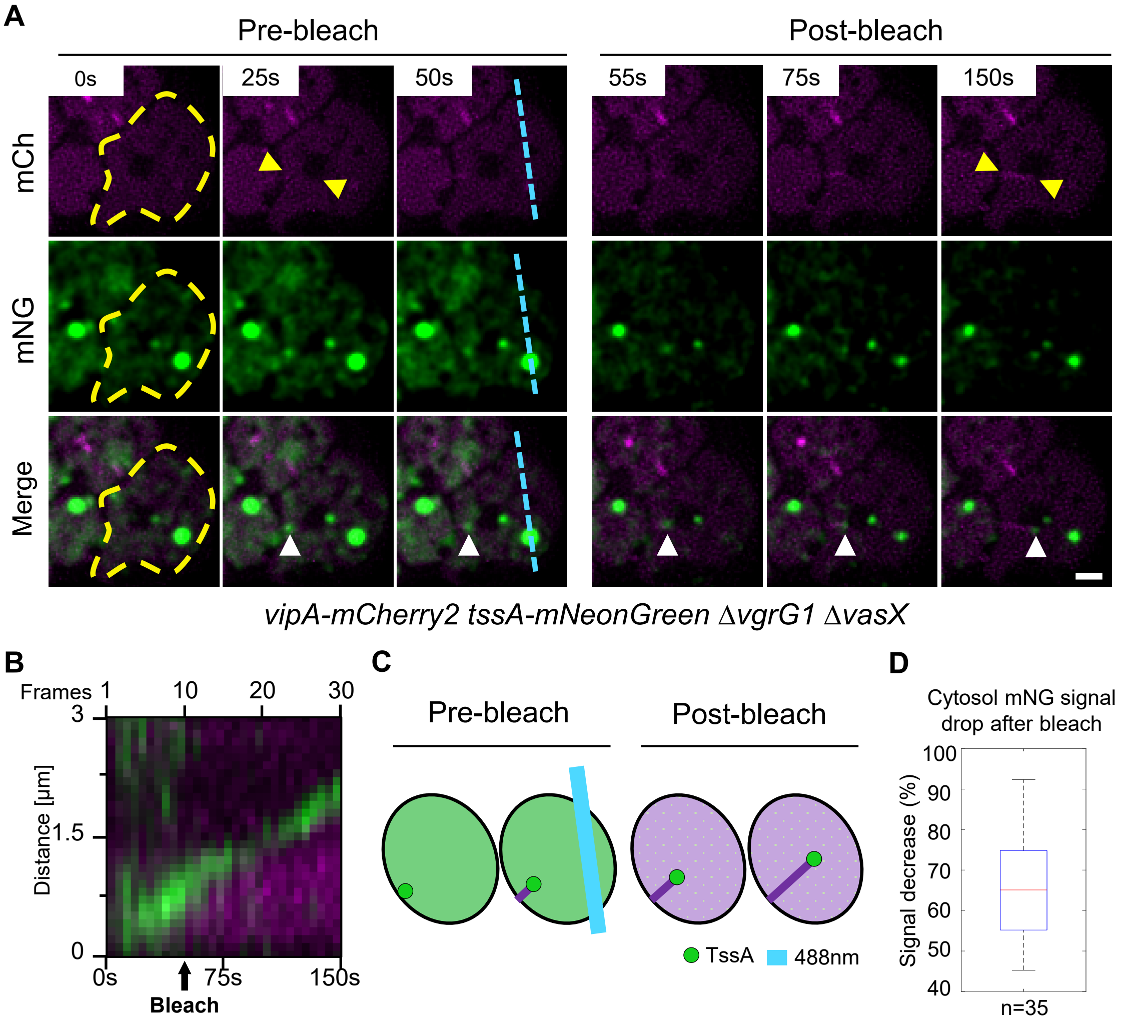

Appendix Figure S1. Photobleaching of cytosolic TssA during sheath polymerization

A. Photobleaching of cytosol (blue dotted line) reveals stable $TssA_{VC}$-mNeonGreen complex (white arrow) at the leading edge of the polymerizing sheath (yellow arrows). Spheroplast induction in liquid culture was achieved by addition of 500µg/ml Ampicillin. Yellow dotted line corresponds to cell outline, scale bar is 2µm.

B. Kymograph of (a), yellow arrows.

C. Scheme of bleaching experiment.

D. Quantification of cytosolic mNeonGreen signal decrease after photobleaching with 488 nm laser. Data information section. In boxplots shown here, the central mark of each box indicates the median and the bottom and top edges of the box indicate the 25th and 75th percentiles, respectively. The whiskers extend to the most extreme data points not considered outliers, and the outliers are plotted individually using the '+' symbol labeled in red.

Appendix Figure S2

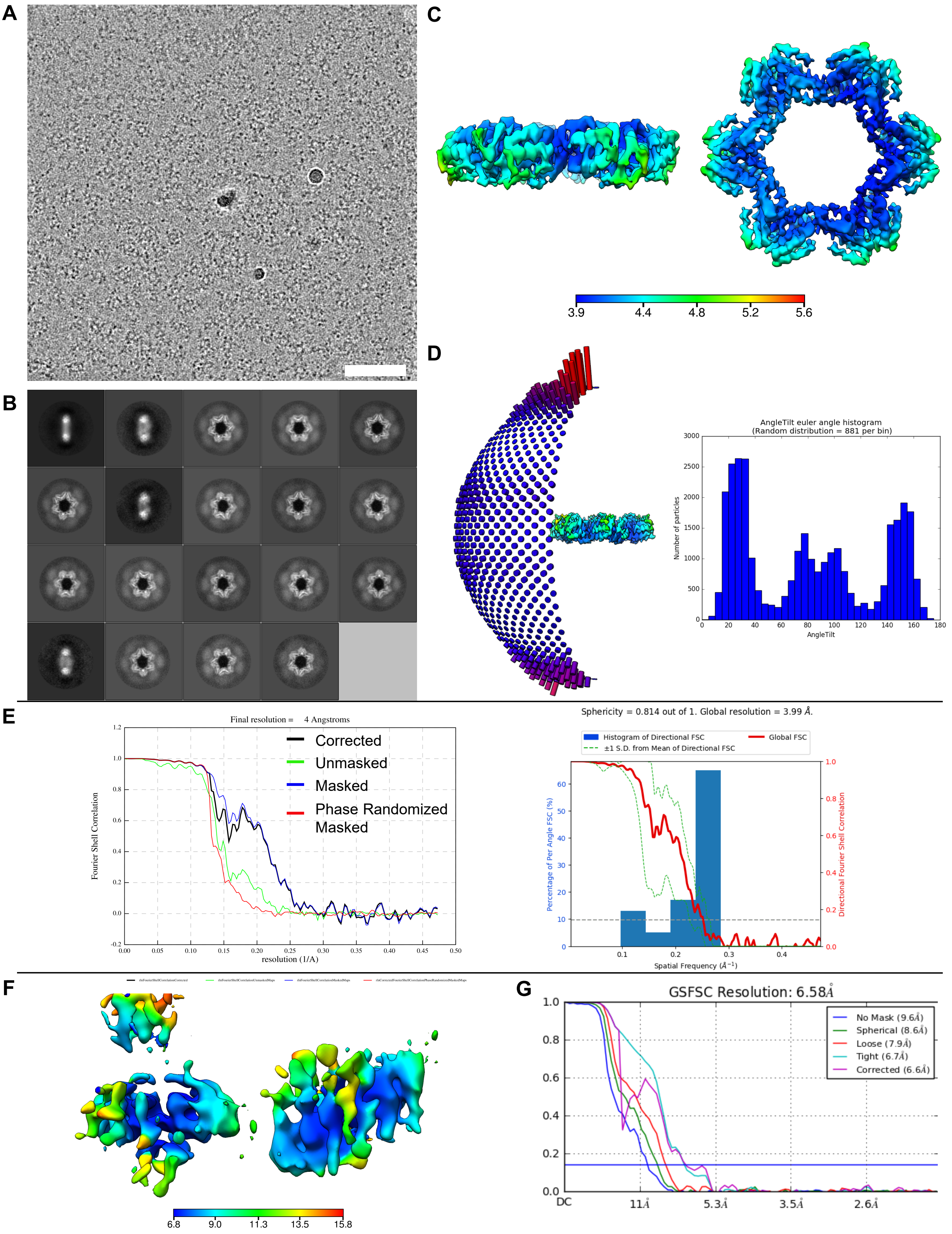

Appendix Figure S2. Cryo-EM of $TssA_{VC}$

A. Cryo-EM image (drift-corrected, dose-weighted and low-pass filtered to 20 Å) of $TssA_{VC}$ sample (scale bar: 50 nm).

B. Representative 2D class averages of $TssA_{VC}$ particles sorted by the number of particles in each class in descending order.

C. Side and top views of $TssA_{VC}$ reconstruction colored according to the local resolution variation, shown in the color bar in Angstroms.

D. Angular distribution plot of $TssA_{VC}$ reconstruction (left), one dimensional tilt angle histogram of TssAVC final reconstruction.

E. FSC curves from RELION postprocessing operation (left) calculated for the unmasked (green) and masked half-maps (blue), the masked half-maps corrected for the influence of the mask (black) and the random-phase corrected half-maps (red). Resolution (4 Å) is measured by the intersection of the FSC curves with the FSC = 0.143 line. Diagnostic output from 3DFSC processing server (right). Global FSC curve (red) and histogram of directional FSC (blue) are shown.

F. Top and side views of Nt2-dimer of $TssA_{VC}$ reconstruction colored according to the local resolution variation, shown in the color bar in Angstroms.

G. FSC curves from cryoSPARC v2 calculated for two unmasked half-maps (blue), after applying a soft spherical (green), soft (red) and tight (cyan) solvent masks, and after applying tight mask with correction by noise substitution (magenta).

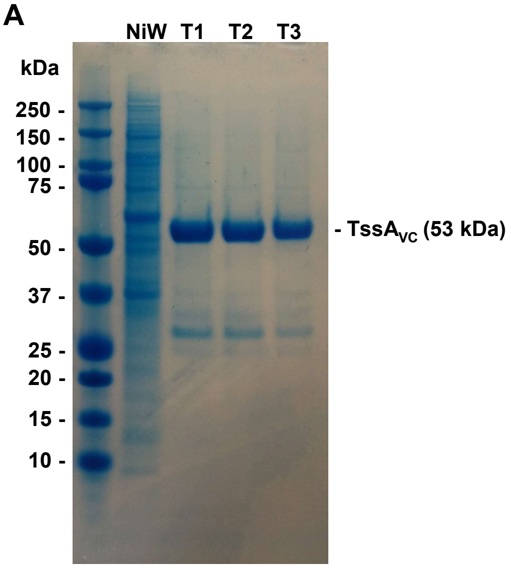

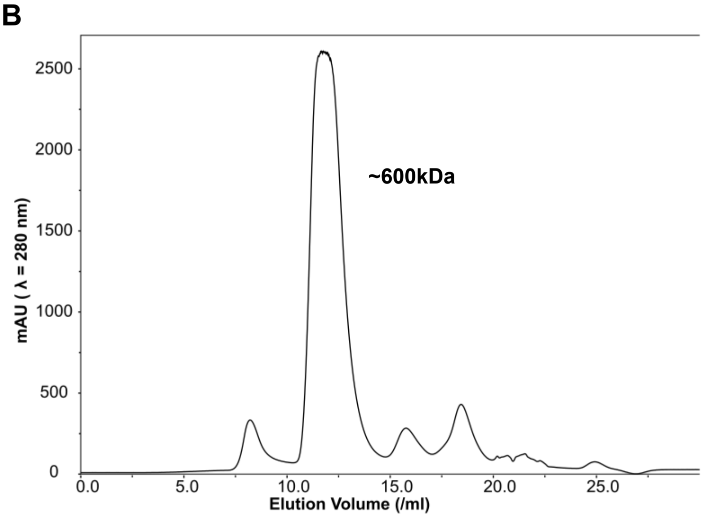

Appendix Figure S3. TssA$_{VC}$ purification

A. Cropped SDS–PAGE of purified TssA$_{VC}$ prep, wash and elute fractions (T1-T3). Detected TssA$_{VC}$ monomer is shown on the right.

B. SEC profile of TssA$_{VC}$ with molecular weight estimation (600kDa).

A

HA-tag pulldown Identified proteins

TssA-HA	TagA-HA
TssA	TagA
ClpV	ClpV
VipB	VipB
TagA	VipA
	TssK

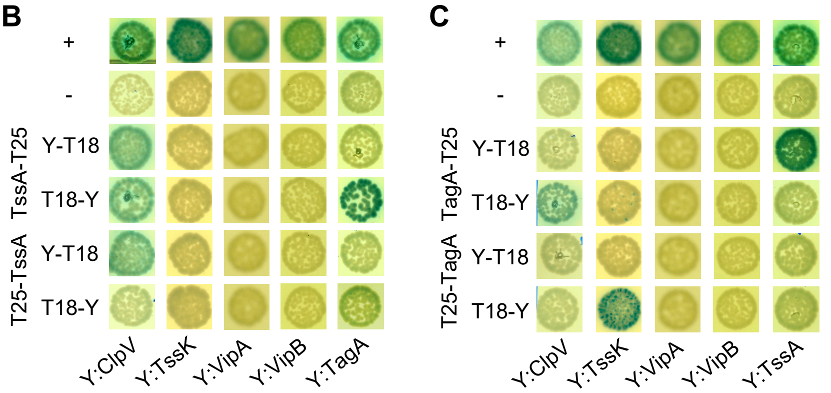

Appendix Figure S4. TssA$_{VC}$ and TagA$_{VC}$ protein interaction assays

A. TssA$_{VC}$ and TagA$_{VC}$ interaction partners found in Co-IP using either TagA$_{VC}$-HA or TagA$_{VC}$-HA as bait.

B. Bacterial two-hybrid assay to test interaction partners found via TssA$_{VC}$-HA Co-IP. TssA$_{VC}$ - ClpV as well as TssA$_{VC}$ - TagA$_{VC}$ tests were positive.

C. Bacterial two-hybrid assay to test interaction partners found via TagA$_{VC}$-HA Co-IP. TagA$_{VC}$ - TssK, TagA$_{VC}$ - ClpV and TagA$_{VC}$ - TssA$_{VC}$ tests were positive. pKT25-zip and pUT18C-zip served as positive control (+), empty vectors pKT25 and pUT18C as negative control (-).

TssA proteins % pairwise identity (% pairwise similarity)

	$TssA_{EC}$	$TssA_{AH}$	$TssA2_{PA}$	$TssA_{BC}$	$TagA_{EC}$
$TssA_{VC}$	19,9% (33,5%)	32,8% (52,4%)	21,8% (41%)	-	-
$TssA_{EC}$	-	23,9% (36,2%)	25,2% (39,2%)	-	-
$TssA1_{PA}$	-	-	-	27,2% (40,2%)	-
$TagA_{VC}$	-	-	-	-	19,3% (32,7%)

Pairwise sequence alignment was performed using EMBOSS NEEDLE tool (https://www.ebi.ac.uk/Tools/psa/emboss_needle/)

Appendix Table S1: Strains used in this study

Organism	Genotype	Plasmid	Relevant features	Source
V. cholerae 2740-80	lacZ$^-$, Str^r, *vipA-mCherry2*		C-terminal chromosomal fusion of *mCherry2* to *vipA*	this study
	lacZ$^-$, Str^r, Δ*tssA, vipA-mCherry2*		*tssA* deletion in *vipA-mCherry2* background	this study
	lacZ$^-$, Str^r, Δ*tagA, vipA-mCherry2*		*tagA* deletion in *vipA-mCherry2* background	this study
	lacZ$^-$, Str^r, Δ*tssA* Δ*tagA, vipA-mCherry2*		*tssA tagA* double deletion in *vipA-mCherry2* background	this study
	lacZ$^-$, Str^r, Δ*vipA*		*vipA* deletion in wild type background	Basler et al., 2012
	lacZ$^-$, Str^r, Δ*tssE*		*tssE* deletion in wild type background	Basler et al., 2012
	lacZ$^-$, Str^r, Δ*tssE* Δ*tagA, vipA-mCherry2*		*tssE tagA* double deletion in *vipA-mCherry2* background	this study
	lacZ$^-$, Str^r, Δ*tssA, vipA-mCherry2*	pBAD24-*tssA*	Complementation of *tssA* deletion in *vipA-mCherry2* background from inducible vector; Amp^r	this study
	lacZ$^-$, Str^r, Δ*tagA, vipA-mCherry2*	pBAD24-*tagA*	Complementation of *tagA* deletion in *vipA-mCherry2* background from inducible vector; Amp^r	this study
	lacZ$^-$, Str^r, Δ*tssA, vipA-mCherry2*	pBAD24-*tssA-mNeonGreen*	Complementation of *tssA* deletion in *vipA-mCherry2* background from inducible vector; Amp^r	this study
	lacZ$^-$, Str^r, Δ*tagA, vipA-mCherry2*	pBAD24-*tagA-mNeonGreen*	Complementation of *tagA* deletion in *vipA-mCherry2* background from inducible vector; Amp^r	this study
	lacZ$^-$, Str^r, *vipA-mCherry2, tssA-mNeonGreen*		C-terminal chromosomal fusion of *mNeonGreen* to *tssA*	this study
	lacZ$^-$, Str^r, *vipA-mCherry2, tagA-mNeonGreen*		C-terminal chromosomal fusion of *mNeonGreen* to *tagA*	this study
	lacZ$^-$, Str^r, Δ*tagA ,vipA-mCherry2, tssA-mNeonGreen*		*tagA* deletion in *vipA-mCherry2 tssA-mNeonGreen* background	this study
	lacZ$^-$, Str^r, *vipA-mCherry2, tssA-mNeonGreen*	pBAD24-*tagA*	Overproduction of TagA in VipA-mCherry2 TssA-mNeonGreen background, Amp^r	this study
	lacZ$^-$, Str^r, Δ*tssA, vipA-mCherry2*	pBAD24-*tssA-HA*	Co-IP, Amp^r	this study
	lacZ$^-$, Str^r, Δ*tagA, vipA-mCherry2*	pBAD24-*tagA-HA*	Co-IP, Amp^r	this study
	lacZ$^-$, Str^r, *lacZ::3xlacO*		3x*lacO* array integrated into *lacZ* gene	this study

Organism	Genotype	Plasmid	Relevant features	Source
	lacZ-, Str^r, *lacZ::6xlacO*		6x*lacO* array integrated into *lacZ* gene	this study
	lacZ-, Str^r, *lacZ::12xlacO*		6x*lacO* array integrated into *lacZ* gene	this study
	lacZ-, Str^r, *lacZ::3xlacO*	pBAD24 *lacI-mNeonGreen*	Fluorescence quantification, generation of mNeonGreen spot with 6 molecules	this study
	lacZ-, Str^r, *lacZ::6xlacO*	pBAD24 *lacI-mNeonGreen*	Fluorescence quantification, generation of mNeonGreen spot with 12 molecules	this study
	lacZ-, Str^r, *lacZ::12xlacO*	pBAD24 *lacI-mNeonGreen*	Fluorescence quantification, generation of mNeonGreen spot with 24 molecules	this study
	lacZ-, Str^r, *ΔvgrG1, ΔvasX, vipA-mCherry2, tssA-mNeonGreen*		*ΔvgrG1, ΔvasX* deletion in double labaled strain used for photobleaching experiments	this study
P. aeruginosa PAO1	Irg^r, *ΔretS, TssB1-mCherry2*		C-terminal chromosomal fusion of *mCherry2* to *tssB1*	this study
	Irg^r, *ΔretS, ΔtssA1, TssB1-mCherry2*		*tssA1* deletion in *tssb1-mCherry2* background	this study
	Irg^r, *ΔretS, ΔtssA1, TssB1-mCherry2*	pPSV35 *tssA1*	Complementation of *tssA1 mutant* from IPTG inducible vector, Gent^R	this study
	Irg^r, *ΔretS, ΔtssA1,TssB1-mCherry2*	pPSV35 *tssA1-mNeonGreen*	Complementation of *tssA1 mutant with tssA1-mNeonGreen* fusion from IPTG inducible vector, Gent^R	this study
	Irg^r, *ΔretS, TssB1-mCherry2*	pPSV35 *tssA1-mNeonGreen*	Ectopic expression of *tssA1-mNeonGreen* fusion from IPTG inducible vector, Gent^R	this study
	Irg^r, *ΔretS, TssB2-mCherry2*		C-terminal chromosomal fusion of *mCherry2* to *tssB2*	this study
	Irg^r, *ΔretS, ΔtssA2, TssB2-mCherry2*		*tssA2* deletion in *tssb2-mCherry2* background	this study
	Irg^r, *ΔretS, TssB2-mCherry2, TssA2-mNeonGreen*		C-terminal chromosomal fusion of *mNeonGreen* to *tssA2* in *tssB-mCherry2 background*	this study
E. coli SM10 λ pir	Km^r, thi-1, thr, leu, tonA, lacY, supE, recA::RP4-2-Tc::Mu, pir	pWM91	Allelic replacement vector used for all in-frame deletions by conjugation; *sacB*, Amp^r	
SM10 λ pir	Km^r, thi-1, thr, leu, tonA, lacY, supE, recA::RP4-2-Tc::Mu, pir	pEXG2	Allelic replacement vector used for all in-frame deletions by conjugation; *sacB*, Gent^r	

Appendix Table S2

Organism	Genotype	Plasmid	Relevant features	Source
DH5α λ pir	F^-, endA1, glnV44, thi-1, recA1, relA1, gyrA96 deoR, nupG, Φ80d*lacZ*ΔM15, (*lacZYA-argF*)U169, hsdR17(rK^- mK^+), λ^-		Cloning strain	
MG1655	F^-, $lambda^-$, rph-1		Bacterial competition assay	
MG1655	F^-, $lambda^-$, rph-1	pBAD24	Bacterial competition assay	

Appendix Table S3. Summary of Co-IP and subsequent MS analysis. (Semi-)quantitative comparison of pulldown experiments with HA-tagged versions of TssA or TagA and WT control. QV—Quantitative value (normalized total spectra), US—exclusive unique spectra (threshold was set to 5). Percent coverage (percentage of all the amino acids in the protein sequence that were covered by identified peptides detected in the sample) threshold was set to 10%.

	**WT**			**TssA-HA**			**TagA-HA**		
**Identified Proteins**	**E1** QV (US)*	**E2** QV (US)*	**E3** QV (US)*	**E1** QV (US)*	**E2** QV (US)*	**E3** QV (US)*	**E1** QV (US)*	**E2** QV (US)*	**E3** QV (US)*
**TssA**	-	-	-	72 (30)	63 (38)	73 (22)	-	-	-
**TagA**	-	-	-	-	3 (5)	8 (5)	50 (28)	15 (19)	8 (16)
**ClpV**	-	13 (7)	-	13 (14)	21 (22)	24 (8)	20 (24)	24 (33)	23 (44)
**VipB**	-	9 (7)	-	7 (6)	8 (8)	-	8 (9)	7 (9)	7 (13)
**VipA**	-	-	-	6 (6)	-	-	6 (7)	4 (6)	3 (6)
**TssK**	-	-	-	-	-	-	5 (6)	7 (9)	5 (10)

E1 – E3 = Experiment 1-3. One experiment contains the average of two to four technical replicates.

*QV = (Average of the spectrum counts for all of the samples) * (Spectrum counts in each sample) / (Individual sample's sum)

*US = Number of unique spectra attributed to a single protein.

**Data collection and processing**		
Magnification	130kx	
Voltage (kV)	300	
Frames (no.)	40	
Electron exposure (e– Å−2)	60	
Defocus range (μm)	[-1:-3.5]	
Pixel size (Å)	1.058	
Symmetry imposed	C6	
Movies (no.)	2963	
Initial particle images (no.)	261412	
Final particle images (no.)	31781	
Map resolution (Å)	3.9	
FSC threshold	0.143	
	**Model**	
**Composition**		
Chains	2	
Non-hydrogen atoms	2888	
Protein residues	182	
Water	0	
Ligands	0	
**Bonds (RMSD)**		
Length (Å) (# > 4σ)	0.006 (0)	
Angles (°) (# > 4σ)	1.324 (6)	
**MolProbity score**	1.78	
**Clash score**	5.19	
**Ramachandran plot (%)**		
Outliers	0	
Allowed	8.43	
Favored	91.57	
**Rotamer outliers (%)**	0	
**Cβ outliers (%)**	0	
**Peptide plane (%)**		
Cis proline/general	0.0/0.0	
Twisted proline/general	0.0/0.0	
**CaBLAM outliers (%)**	3.45	
**ADP (B-factors)**		
Iso/Aniso (#)	1454/0	
min/max/mean		
Protein	91.97/163.67/119.96	
Nucleotide	---	
Ligand	---	
Water	---	
**Occupancy**		
Mean	1	
occ = 1 (%)	100	
0 < occ < 1 (%)	0	
occ > 1 (%)	0	
	**Data**	
**Box**		
Lengths (Å)	69.83, 47.61, 49.73	
Angles (°)	90.00, 90.00, 90.00	
**Supplied Resolution (Å)**	3.9	
**Resolution Estimates (Å)**	**Masked**	**Unmasked**
d FSC (half maps; 0.143)	3.6	2
d 99 (full/half1/half2)	4.1/6.8/7.0	3.9/5.2/5.2
d model	3.9	3.9
d FSC model (0/0.143/0.5)	3.4/3.7/7.2	3.4/3.8/13.9
**Map min/max/mean**	-19.10/37.15/-0.00	
	**Model vs. Data**	
CC (mask)	0.68	
CC (box)	0.52	
CC (peaks)	0.23	
CC (volume)	0.68	
Mean CC for ligands	---	
