# Supplementary material for: Diverse roles of TssA‐like proteins in the assembly of bacterial type VI secretion systems
Source: EMBO J. 2019 Aug 12;38(18):e100825. doi: 10.15252/embj.2018100825 (PMC6745524; doi:10.15252/embj.2018100825)
Supplement: Supplementary file 2 — Expanded View Figures PDF [file EMBJ-38-e100825-s002.pdf]

Expanded View Figures

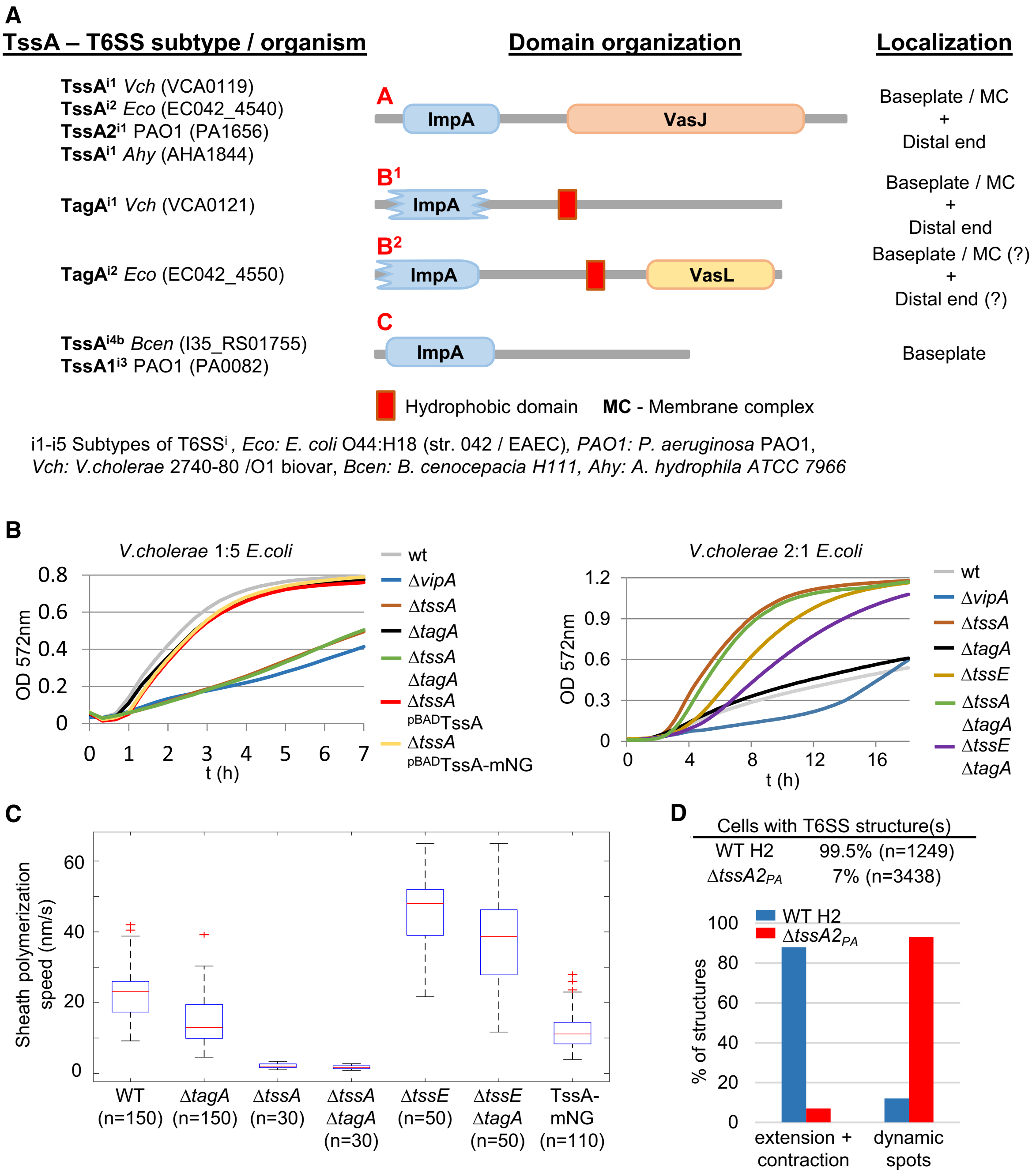

Figure EV1.

© 2019 The Authors

Figure EV1. TssA protein domain organization and mutant phenotypes.

A Protein domain organization and proposed subcellular localization of *V. cholerae* 2,740–80/O1, *P. aeruginosa* PAO1, *E. coli* O44:H18 (str. 042/EAEC), *A. hydrophila* ATCC7966, and *B. cenocepacia* H111 TssA proteins. TssA proteins display three specific protein domain architectures A, B^1/B^2, and C. Most common architecture of each class is shown.

B Bacterial competition assay comparing T6SS-dependent killing efficiency in different strain backgrounds. Fast killing kinetics via T6SS can be best observed by mixing strains in a ratio of 1:5 (*V. cholerae* : *E. coli*) (left panel), while less efficient or slow killing kinetics can be observed by mixing strains in a ratio of 2:1 (*V. cholerae*: *E. coli*) (right panel). Δ*tssA* mutant kills prey *E. coli* cells at a very slow rate, comparable to a Δ*tssE* mutant strain (right panel). Δ*tagA* mutant shows similar killing kinetics to WT (left and right panel). Δ*tagA* Δ*tssE* double mutant shows a shift in killing kinetics compared to Δ*tagA* mutant.

C T6SS sheath polymerization speed in WT and different mutant backgrounds. Polymerization speed in the Δ*tagA* mutant is reduced (13 nm/s) compared to WT speed (23 nm/s) and drastically reduced in the Δ*tssA* mutant (3 nm/s). Polymerization speed in the Δ*tssE* mutant is 48 nm/s and decreases to 38 nm/s in the Δ*tagA* Δ*tssE* double mutant. The strain harboring $TssA_{VC}$-mNeonGreen fusion displays decreased polymerization speed (11 nm/s) compared to parental strain without a tag on TssA (23 nm/s).

D Quantification of structures per cell and percentage of dynamic sheath spots versus extending and contracting structures in H2-T6SS WT and Δ$tssA2_{PA}$ strain. Δ$tssA2_{PA}$ strain displays very few structures and mostly dynamic spots.

Data information: In boxplots shown here, the central mark of each box indicates the median and the bottom and top edges of the box indicate the 25th and 75th percentiles, respectively. The whiskers extend to the most extreme data points not considered outliers, and the outliers are plotted individually using the "+" symbol labeled in red.

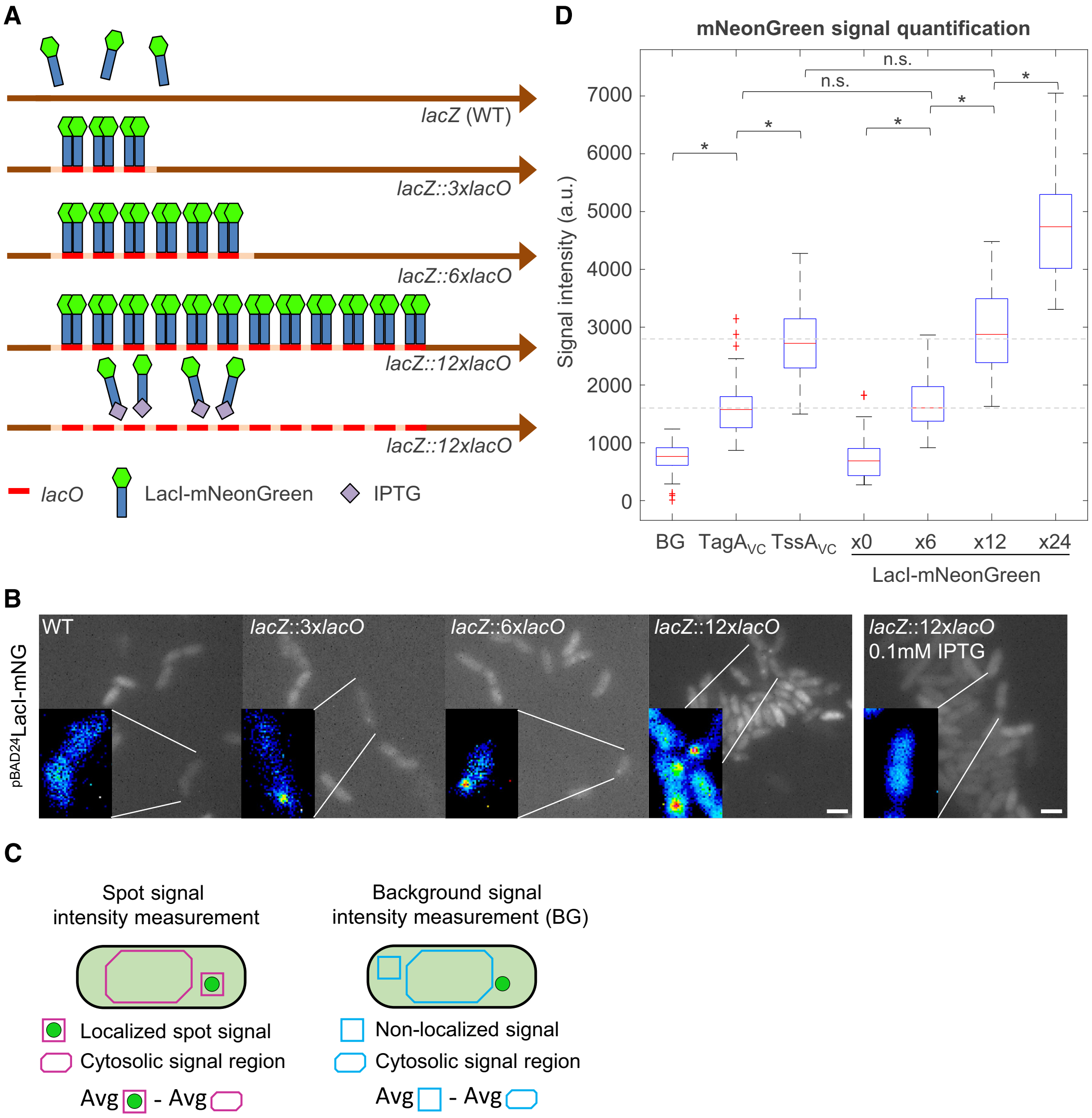

Figure EV2. Fluorescence quantification.

A Scheme of *lacO* array integration into *V. cholerae* chromosome at the *lacZ* locus. 3, 6, or 12 copies of the *lacO* operator accommodate 6, 12, or 24 molecules of LacI-mNeonGreen, respectively. IPTG (isopropyl-β-D-thiogalactopyranoside) binds to the *lac* repressor LacI and disrupts its ability to bind DNA.

B Fluorescence microscopy of strains harboring different *lacO* arrays. LacI-mNeonGreen was expressed from pBAD24 plasmid by induction with 0.002% L-arabinose. Images depicted for each strain are summed stacks of a short time lapse series. Inlays show magnified regions of interest, false colored with rainbow LUT using ImageJ. Cells contained one or two discrete foci of LacI-mNeonGreen fluorescence. Scale bars: 2 µm.

C Scheme of cells harboring a mNeonGreen protein fusion and quantification procedure. Raw mNeonGreen signal was collected using ImageJ. Spot fluorescence signal (either localized signal of a complex or non-localized signal) of three consecutive frames (red or blue square, approximately 5 × 5 pixels) in a cell was averaged. An average signal of the cell cytosol (red or blue corner rectangle) of the corresponding three frames was then subtracted from the spot signal. This resulted in fluorescence signal intensity of a spot (red square) or background signal intensity (blue square).

D Quantification of mNeonGreen fluorescence signals. Distributions of signal intensities measured in raw data for each strain are represented in the graph. 0× LacI-mNeonGreen corresponds to the parental strain (WT), which does not harbor a *lacO* array. Signal distributions of 6×, 12×, and 24× LacI-mNeonGreen correspond to strains harboring 3x*lacO*, 6x*lacO*, and 12x*lacO* array, respectively. Distribution of signal intensities for $TagA_{VC}$ corresponds to the strain harboring $TagA_{VC}$-mNeonGreen chromosomal fusion. Distribution of signal intensities for $TssA_{VC}$ corresponds to the strain harboring $TssA_{VC}$-mNeonGreen chromosomal fusion. BG corresponds to cytosolic mNeonGreen background signal in the strain harboring $TssA_{VC}$-mNeonGreen chromosomal fusion. BG (n = 41), $TagA_{VC}$ (n = 111), $TssA_{VC}$ (n = 76), x0 (n = 44), x3 (n = 76), x6 (n = 62), x12 (n = 88). n.s.: Distributions are not significantly different at alpha = 0.005 (two-sample Kolmogorov–Smirnov test). * distributions are significantly different from each other, $P < 0.001$ (P-values Bonferroni corrected).

Data information: In boxplots shown here, the central mark of each box indicates the median and the bottom and top edges of the box indicate the 25th and 75th percentiles, respectively. The whiskers extend to the most extreme data points not considered outliers, and the outliers are plotted individually using the "+" symbol labeled in red.

 © 2019 The Authors

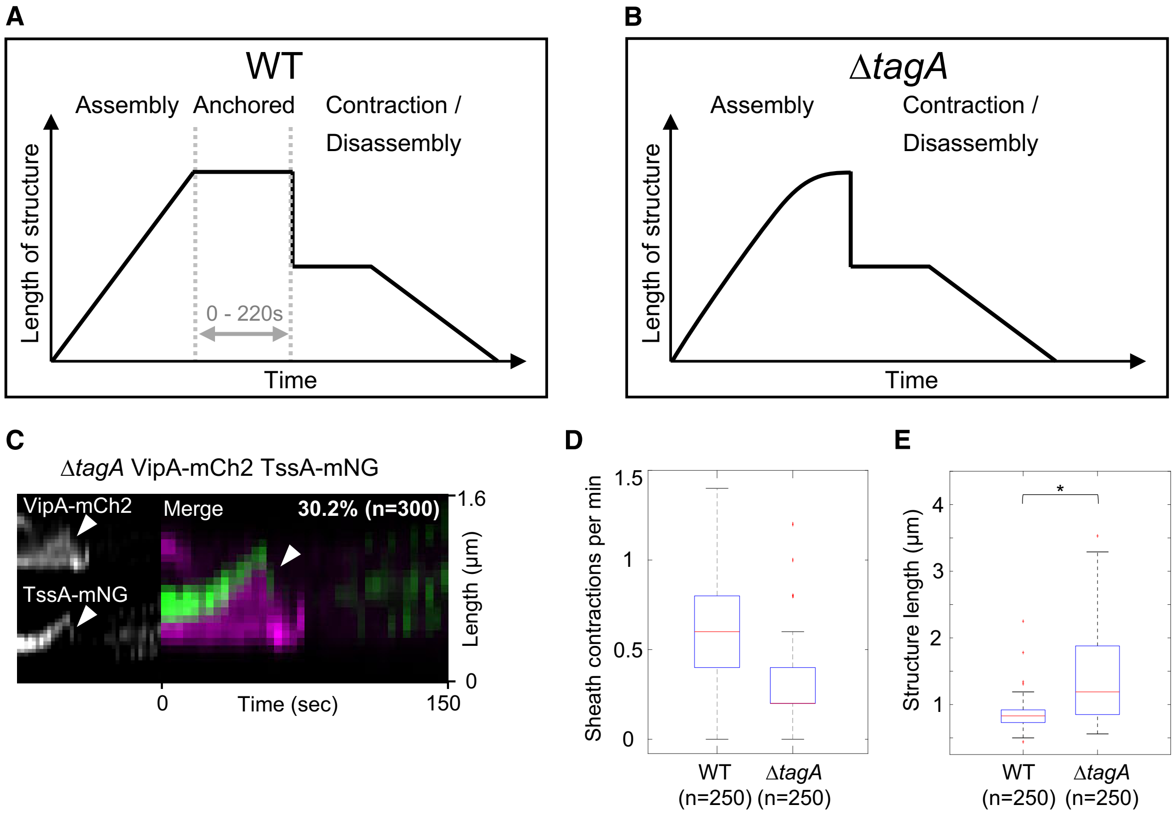

Figure EV3. $TagA_{VC}$ effect on sheath and $TssA_{VC}$ dynamics.

A Representative scheme of T6SS sheath dynamics in wild type. T6SS sheath stays extended or presumably anchored to the membrane via TagA for up to 220 s before contraction (average anchored time: 50 s, n = 150).

B Representative scheme of T6SS sheath dynamics in the *tagA* mutant. Sheath does not stay extended before contraction.

C Representative kymograph of VipA-mCherry2 and TssA-mNeonGreen dynamics in a *tagA* mutant strain background. 30% of all sheath structures show TssA-mNeonGreen signal at distal end (white arrows) after contraction.

D Quantification of number of sheath contractions. 0.6 sheath contractions per minute per cell were measured in WT and 0.2 contractions per minute per cell in the Δ*tagA* mutant.

E Sheath length significantly increases in the Δ*tagA* mutant. * Samples are significantly different from each other (two-sample *t*-test, $P < 0.001$).

Data information: In boxplots shown here, the central mark of each box indicates the median and the bottom and top edges of the box indicate the 25th and 75th percentiles, respectively. The whiskers extend to the most extreme data points not considered outliers, and the outliers are plotted individually using the "+" symbol labeled in red.

© 2019 The Authors

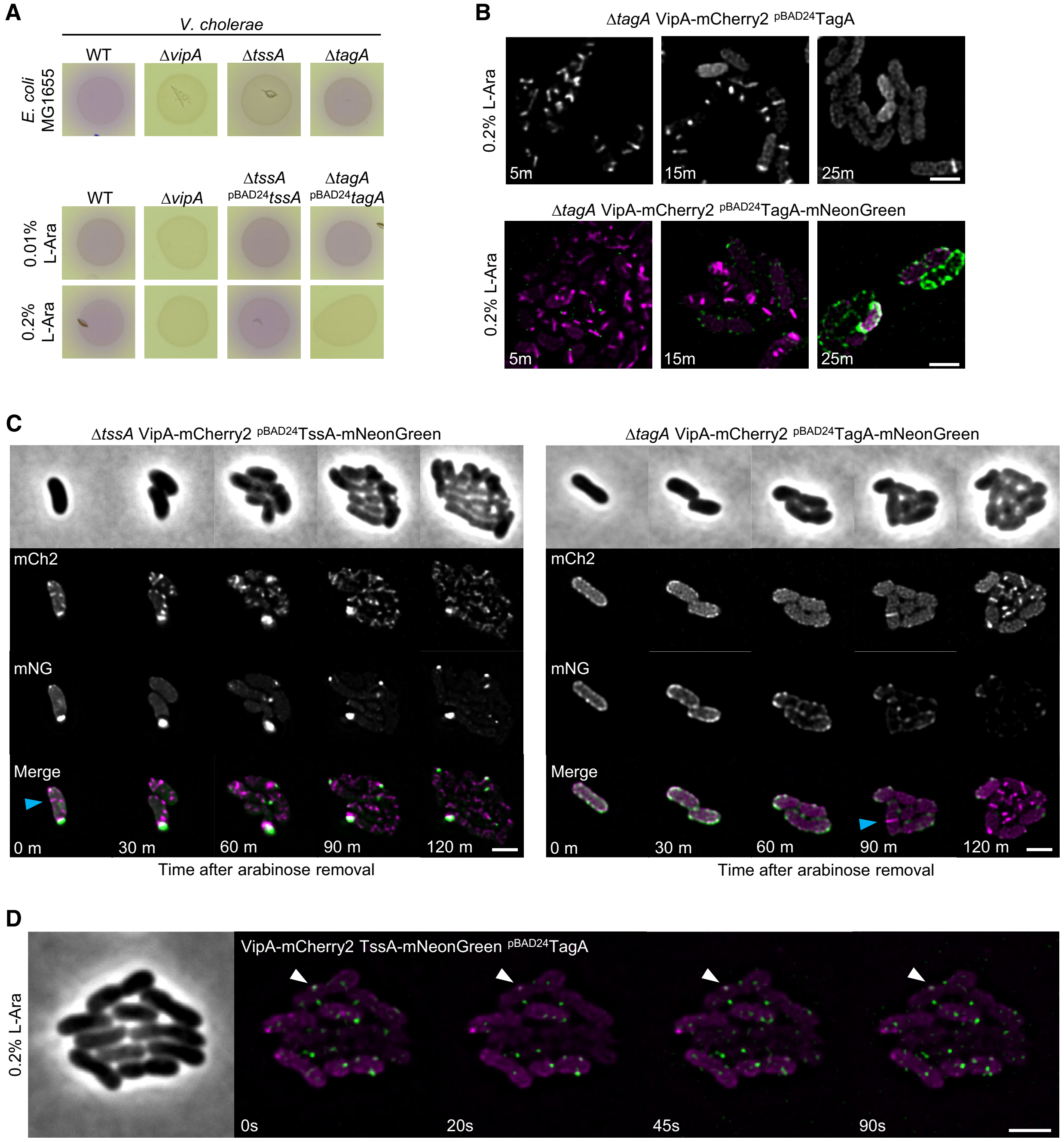

Figure EV4. Overproduction of TagA inhibits T6SS in *V. cholerae*.

A Bacterial competition assay. Killing of *E. coli* (MG1655, *lacZ*+) by *V. cholerae* T6SS can be observed as CPRG substrate is converted by released β-galactosidase over time (red color). *ΔtssA*$_{VC}$ strain shows significantly decreased killing efficiency, while *tagA*$_{VC}$ knockout is indistinguishable from WT killing efficiency. *ΔvipA* strain was used as T6SS-negative control (upper panel). Overproduction of TagA$_{VC}$ but not TssA$_{VC}$ leads to inhibition of T6SS-mediated killing (lower panel).

B Overproduction of TagA$_{VC}$ and TagA$_{VC}$-mNeonGreen leads to inhibition of T6SS sheath formation. Overproduced TagA$_{VC}$-mNeonGreen localized predominantly at the cell periphery in discrete foci. Scale bars: 2 μm.

C Indicated strains were pre-induced with 0.2% L-arabinose for 1 h, then washed, and observed using fluorescence microscopy. Dynamic structures (blue arrow) can be detected directly after washing in the TssA$_{VC}$-mNeonGreen overproducing strain (left panel). The strain overproducing TagA$_{VC}$-mNeonGreen was only able to form T6SS structures after 90-min recovery period (right panel). Scale bar: 2 μm.

D Time lapse series of a VipA-mCherry2 TssA$_{VC}$-mNeonGreen double-tagged strain overproducing TagA$_{VC}$ from plasmid. TagA$_{VC}$ expression was induced for 1 h with 0.2% L-arabinose prior to imaging. TssA$_{VC}$-mNeonGreen displays non-dynamic behavior and co-localizes with sheath foci in the cell periphery (white arrow). Scale bar: 2 μm.

 © 2019 The Authors

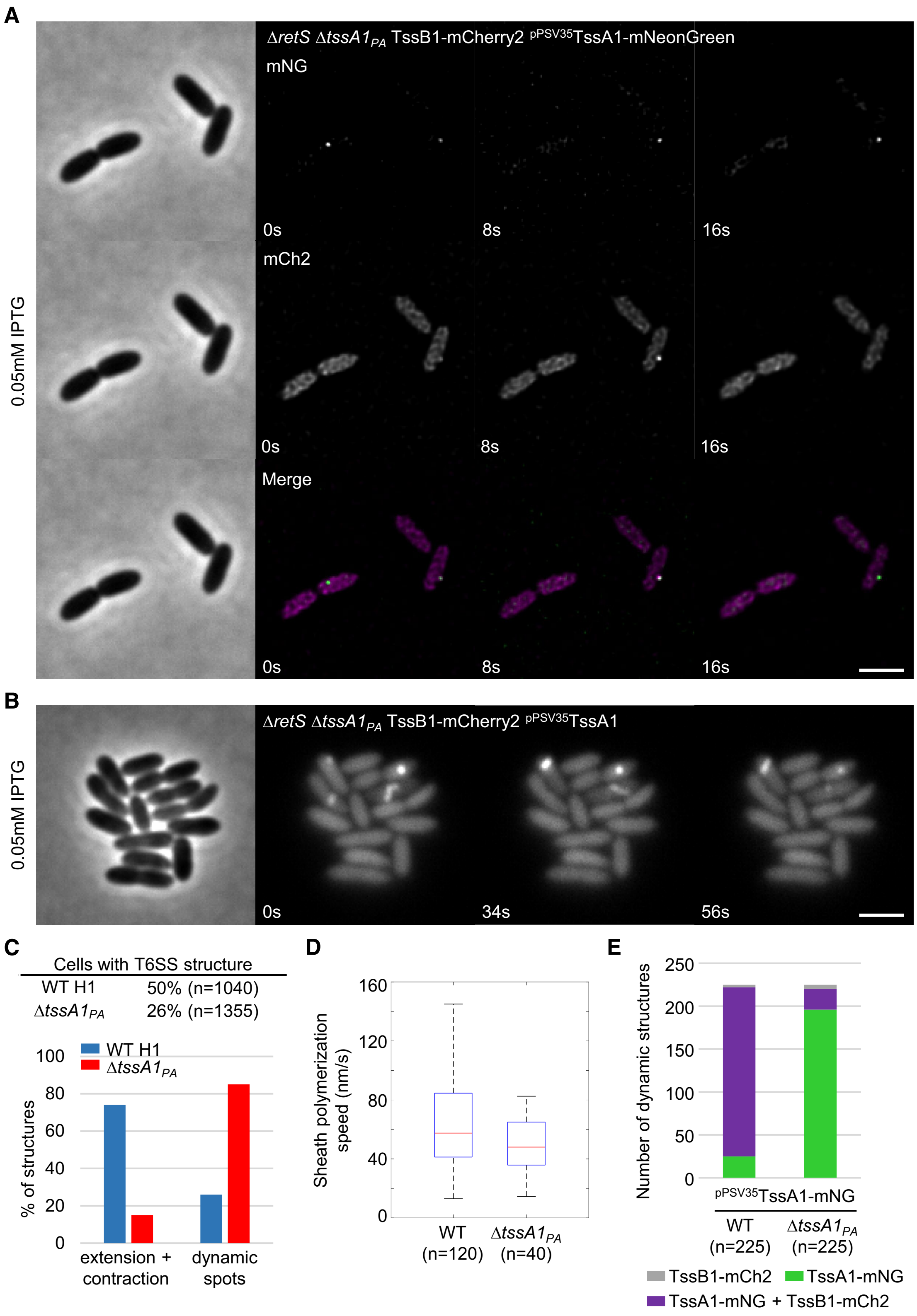
A
ΔretS ΔtssA1PA TssB1-mCherry2 pPSV35TssA1-mNeonGreen
mNG
0s
8s
16s
mCh2
Merge
0.05mM IPTG
B
ΔretS ΔtssA1PA TssB1-mCherry2 pPSV35TssA1
34s
56s
C
Cells with T6SS structure
WT H1 50% (n=1040)
ΔtssA1PA 26% (n=1355)
WT H1
ΔtssA1PA
% of structures
extension + contraction
dynamic spots
D
Sheath polymerization speed (nm/s)
WT (n=120)
ΔtssA1PA (n=40)
E
Number of dynamic structures
pPSV35TssA1-mNG
WT (n=225)
ΔtssA1PA (n=225)
TssB1-mCh2
TssA1-mNG
TssA1-mNG + TssB1-mCh2

Figure EV5.

Figure EV5. TssA1$_{PA}$ complementation and quantification of dynamic sheaths.

A Time lapse fluorescence microscopy of Δ*retS* Δ*tssA1$_{PA}$* TssB1-mCherry2 strain harboring pPSV35 plasmid with TssA1$_{PA}$-mNeonGreen fusion. No dynamic T6SS sheath structures can be observed. Scale bar: 2 μm.

B Time lapse series of Δ*retS* Δ*tssA1* TssB1-mCherry2 strain harboring pPSV35 plasmid with TssA1$_{PA}$. Δ*tssA1* mutation can be complemented with native copy of TssA1 expressed from pPSV35 plasmid. Several dynamic sheath structures can be observed. Scale bar: 2 μm.

C Quantification of structures per cell and percentage of dynamic sheath spots versus extending and contracting structures. Δ*tssA1$_{PA}$* strain displays less structures and mostly dynamic spots.

D Polymerization speed of T6SS sheath in Δ*retS* TssB1-mCherry2 (WT) and Δ*retS* Δ*tssA1$_{PA}$* TssB1-mCherry2 strains. Polymerization speed is 58 nm/s on average in WT and 48 nm/s on average in the Δ*tssA1$_{PA}$* mutant.

E Colocalization of TssA1$_{PA}$-mNeonGreen with TssB1 sheath during T6SS dynamics. WT, Δ*retS* TssB1-mCherry2 strain harboring pPSV35 plasmid with TssA1$_{PA}$-mNeonGreen fusion. Δ*tssA1$_{PA}$*, Δ*retS* Δ*tssA1$_{PA}$* TssB1-mCherry2 strain harboring pPSV35 plasmid with TssA1$_{PA}$-mNeonGreen fusion. Proportion of dynamic structures consisting of TssA1$_{PA}$-mNeonGreen spots that colocalize with sheath structures and spots (WT) or spots (Δ*tssA1$_{PA}$*) is labeled in magenta. Fraction of dynamic TssA1$_{PA}$-mNeonGreen spots without sheath signal in close proximity is labeled in green. Percentage of sheath signals (spots or structures) without TssA1$_{PA}$-mNeonGreen spot in proximity is labeled in gray.

Data information section. In boxplots shown here, the central mark of each box indicates the median and the bottom and top edges of the box indicate the 25th and 75th percentiles, respectively. The whiskers extend to the most extreme data points not considered outliers.

© 2019 The Authors
