# Supplementary material for: Diverse roles of TssA‐like proteins in the assembly of bacterial type VI secretion systems
Source: EMBO J. 2019 Aug 12;38(18):e100825. doi: 10.15252/embj.2018100825 (PMC6745524; doi:10.15252/embj.2018100825)
Supplement: Supplementary file 3 — Movie EV1 [file EMBJ-38-e100825-s003.zip › EMBOJ-2018-100825R_MovieEV1.rtf]

EMBOJ-2018-100825R_MovieEV1.
Time lapse series of T6SS activity in parental strain (VipA-mCherry2) and ∆tssA mutant background. Images were acquired every 5 seconds (parental strain) or every 10 seconds (∆tssA mutant). Movie plays at 10 frames per second. Scale bars are 2 µm. 
