# Supplementary material for: Diverse roles of TssA‐like proteins in the assembly of bacterial type VI secretion systems
Source: EMBO J. 2019 Aug 12;38(18):e100825. doi: 10.15252/embj.2018100825 (PMC6745524; doi:10.15252/embj.2018100825)
Supplement: Supplementary file 4 — Movie EV2 [file EMBJ-38-e100825-s004.zip › EMBOJ-2018-100825R_MovieEV2.rtf]

EMBOJ-2018-100825R_MovieEV2.
H2 T6SS dynamics in parental strain (∆retS TssB2-mCherry2) and ÄtssA2PA strain. Images were acquired every 20 seconds (parental strain) or every 25 seconds (∆tssA2PA mutant). Movie plays at 10 frames per second. Scale bars are 2 µm. 
