# Supplementary material for: Diverse roles of TssA‐like proteins in the assembly of bacterial type VI secretion systems
Source: EMBO J. 2019 Aug 12;38(18):e100825. doi: 10.15252/embj.2018100825 (PMC6745524; doi:10.15252/embj.2018100825)
Supplement: Supplementary file 6 — Movie EV4 [file EMBJ-38-e100825-s006.zip › EMBOJ-2018-100825R_MovieEV4.rtf]

EMBOJ-2018-100825R_MovieEV4.Photobleaching of cytosolic TssA during sheath polymerization in spheroplasts of ∆grG1 ∆asX VipA-mCherry2 TssAVC-mNeonGreen tagged strain. For every example, ten images were acquired every 5 seconds, followed by photobleaching using a VS-AOTF 488 m Laser (<5 seconds). Twenty more images were acquired every 5 seconds after photobleaching. Deconvolution was applied to both channels. Movie plays at 10 frames per second. Scale bars are 4 µm.
