# Supplementary material for: Diverse roles of TssA‐like proteins in the assembly of bacterial type VI secretion systems
Source: EMBO J. 2019 Aug 12;38(18):e100825. doi: 10.15252/embj.2018100825 (PMC6745524; doi:10.15252/embj.2018100825)
Supplement: Supplementary file 7 — Movie EV5 [file EMBJ-38-e100825-s007.zip › EMBOJ-2018-100825R_MovieEV5.rtf]

EMBOJ-2018-100825R_MovieEV5.Fluorescence microscopy of T6SS dynamics in the ∆etS TssB2-mCherry2 TssA2PA-mNeonGreen strain. Images were acquired every 15 seconds and deconvolution was applied to both channels. Movie plays at 10 frames per second. Scale bar is 1 µm.
