# Supplementary material for: Diverse roles of TssA‐like proteins in the assembly of bacterial type VI secretion systems
Source: EMBO J. 2019 Aug 12;38(18):e100825. doi: 10.15252/embj.2018100825 (PMC6745524; doi:10.15252/embj.2018100825)
Supplement: Supplementary file 8 — Movie EV6 [file EMBJ-38-e100825-s008.zip › EMBOJ-2018-100825R_MovieEV6.rtf]

EMBOJ-2018-100825R_MovieEV6.Image series of T6SS activity in ∆agA mutant background. Images were acquired every 5 seconds. Movie plays at 10 frames per second. Scale bar is 2 µm.
