# Supplementary material for: Diverse roles of TssA‐like proteins in the assembly of bacterial type VI secretion systems
Source: EMBO J. 2019 Aug 12;38(18):e100825. doi: 10.15252/embj.2018100825 (PMC6745524; doi:10.15252/embj.2018100825)
Supplement: Supplementary file 10 — Movie EV8 [file EMBJ-38-e100825-s010.zip › EMBOJ-2018-100825R_MovieEV8.rtf]

EMBOJ-2018-100825R_MovieEV8.H1 T6SS dynamics time lapse series in parental strain (∆etS TssB1-mCherry2) and ΔssA1PA strain. Images were acquired every 3 seconds for parental strain and ∆ssA1PA mutant. Movie plays at 10 frames per second. Scale bars are 2 µm.
