# Supplementary material for: Diverse roles of TssA‐like proteins in the assembly of bacterial type VI secretion systems
Source: EMBO J. 2019 Aug 12;38(18):e100825. doi: 10.15252/embj.2018100825 (PMC6745524; doi:10.15252/embj.2018100825)
Supplement: Supplementary file 11 — Movie EV9 [file EMBJ-38-e100825-s011.zip › EMBOJ-2018-100825R_MovieEV9.rtf]

EMBOJ-2018-100825R_MovieEV9.Image series of T6SS dynamics in ∆etS TssB1-mCherry2 strain harboring pPSV35 plasmid with TssA1PA-mNeonGreen fusion. Images were acquired every 4 seconds. Deconvolution was applied to both channels. Movie plays at 10 frames per second. Scale bar is 1 µm.
